# Tumor necrosis as a prognostic variable for the clinical outcome in patients with renal cell carcinoma: a systematic review and meta-analysis

**DOI:** 10.1186/s12885-018-4773-z

**Published:** 2018-09-03

**Authors:** Lijin Zhang, Zhenlei Zha, Wei Qu, Hu Zhao, Jun Yuan, Yejun Feng, Bin Wu

**Affiliations:** 1 Department of Urology, Affiliated Jiang-yin Hospital of the Southeast University Medical College, Jiang-yin, 214400 People’s Republic of China; 2 Department of Pharmacy, Affiliated Jiang-yin Hospital of the Southeast University Medical College, Jiang-yin, 214400 People’s Republic of China

**Keywords:** Renal cell carcinoma, Tumor necrosis, Prognosis, Meta-analysis

## Abstract

**Background:**

Tumor necrosis (TN) correlates with adverse outcomes in numerous solid tumors**.** However, its prognostic value in renal cell carcinoma (RCC) remains unclear. In this study, we performed a meta-analysis to evaluate associations between TN and cancer-specific survival (CSS), overall survival (OS), recurrence-free survival (RFS) and progression-free-survival (PFS) in RCC.

**Methods:**

Electronic searches in PubMed, EMBASE and Web of Science were conducted according to the PRISMA statement. Hazard ratios (HRs) and 95% confidence intervals (95% CIs) were calculated to evaluate relationships between TN and RCC. A fixed- or random-effects model was used to calculate pooled HRs and 95%CIs according to heterogeneity.

**Results:**

A total of 34 cohort studies met the eligibility criteria of this meta-analysis. The results showed that TN was significantly predictive of poorer CSS (HR = 1.37, 95% CI: 1.23–1.53, *p* < 0.001), OS (HR = 1.29, 95% CI: 1.20–1.40, *p* < 0.001), RFS (HR = 1.55, 95% CI: 1.39–1.72, *p* < 0.001) and PFS (HR = 1.31, 95% CI: 1.17–1.46, *p* < 0.001) in patients with RCC. All the findings were robust when stratified by geographical region, pathological type, staging system, number of patients, and median follow-up.

**Conclusions:**

The present study suggests that TN is associated with CSS, OS, RFS and PFS clinical outcomes of RCC patients and may serve as a predictor of poor prognosis in these patients.

**Electronic supplementary material:**

The online version of this article (10.1186/s12885-018-4773-z) contains supplementary material, which is available to authorized users.

## Background

Renal cell carcinoma (RCC), the third most common urologic tumor, accounts for 2–3% of all adult malignancies [1], and its incidence has continuously increased over the past few decades [2]. Although most RCC cases are diagnosed at an early stage, approximately 20% of patients undergoing curative nephrectomy will subsequently develop metastasis during the follow-up period [3]. Due to the varying efficacy of adjuvant therapies in RC, it is necessary to define more prognostic factors that will allow identification of patients at high risk of recurrence who may benefit from such treatment.

Currently, TNM stage classification [4] and the Fuhrman grade system [5] are the most important factors affecting the prognosis of patients with RCC. Additionally, several integrated prognostic models and histologic characteristics have been studied for their prognostic impact, including the American Joint Committee on Cancer (AJCC) staging system [6], International Society of Urologic Pathologists (ISUP) [7] and Mayo Clinic Stage, Size, Grade and Necrosis (SSIGN) Score [8], though these parameters are not entirely reliable. Tumor necrosis (TN) is believed to define regions of severe and chronic hypoxia, and there is renewed interest in using TN to predict prognosis after tumor resection. However, the prognostic impact of TN in RCC remains controversial, and there is increasing debate on whether TN can provide any additional information beyond grade and stage [9].

Hence, to further clarify the prognostic value of TN in RCC, we performed a systematic review and meta-analysis of the available published literature to evaluate whether the presence of TN has a prognostic impact on cancer-specific survival (CSS), overall survival (OS), recurrence-free survival (RFS) and progression-free-survival (PFS) in RCC patients.

## Methods

### Literature search strategy

According to the PRISMA guidelines [10], a comprehensive literature search was conducted using the electronic databases of PubMed, EMBASE and Web of Science up to April 2018. The MeSH terms and full text terms adopted were as follows: “kidney neoplasms”, “renal cell cancer”, “renal cell carcinoma”, “necrosis”, “tumor necrosis”, “prognosis”, “prognostic outcome”, “survival outcome”, “oncologic outcome” and their combinations. We also manually searched the reference lists of reviews, meta-analyses, and selected research articles to identify other “gray literature”. The language of the publications was restricted to English.

### Inclusion and exclusion criteria

Eligible studies were selected only if they met the following criteria: (i) RCC and TN were pathologically confirmed, with all patients undergoing surgical resection; (ii) the potential prognostic value of TN for CSS, OS, RFS and PFS were reported; (iii) the authors categorically reported hazard ratios (HRs) and 95% confidence intervals (95%CIs), or they could be computed from the given data. Studies were excluded if the following criteria were met: (i) animal models or cancer cell lines were used; (ii) reviews, letters, commentaries, case reports and non-original articles; (iii) TN, clinical features and survival outcome were not analyzed; (iv) lacking sufficient data to acquire HRs and 95%CIs; (v) not in English. Additionally, when duplicate articles were found, only the most informative and recent article was adopted.

### Data extraction and quality assessments

Two investigators independently extracted data of eligible studies using a standardized form for the following information: author identification, year of publication, country, period of recruitment, study design, age of patients, gender ratio, sample size, follow-up time, study design, interpretation of TN, histology and survival end point. For HRs and 95% CIs, multivariate analysis data were preferentially adopted. If these data were not available, then univariate analysis of survival outcomes was extracted instead. All discrepancies between the investigators reached a consensus through discussion. The methodological quality of the included cohort studies was assessed using the Newcastle-Ottawa scale (NOS) [11]. Each study was assessed using 8 methodology items in 3 domains with a score ranging from 0 to 9. High scores indicated high quality, a study with a score ≥ 6 was regarded as high quality, a score < 6 was regarded as low quality.

### Statistical analysis

Statistical analyses were performed using Stata 12.0 software (Stat Corp, College Station, TX, USA). Dichotomous variables were calculated using HRs, and pooled HRs with 95% CI were used to evaluate the association of TN with RCC prognosis (CSS, OS, RFS and PFS). A heterogeneity test of the pooled HR was conducted using a Chi-square-based *Q* test and Higgins ***I***^2^ statistic. When ***I***^2^ < 50% or P_heterogeneity_ > 0.1, no obvious heterogeneity existed among the studies, and the fixed-effects (FE) model would be applied; otherwise, the random-effects (RE) model was applied. To obtain a more precise evaluation of heterogeneity, subgroup analysis was performed for CSS, OS and RFS based on geographical region, pathological types, staging system, No. of patients and median follow-up. Publication bias was examined using funnel plots and Egger’s linear regression test. Additionally, sensitivity analysis was used to estimate the robustness of the results via sequential omission of individual studies. A *p* value of < 0.05 was considered to indicate significance.

## Results

### Search and eligible studies

**Fig. 1:**
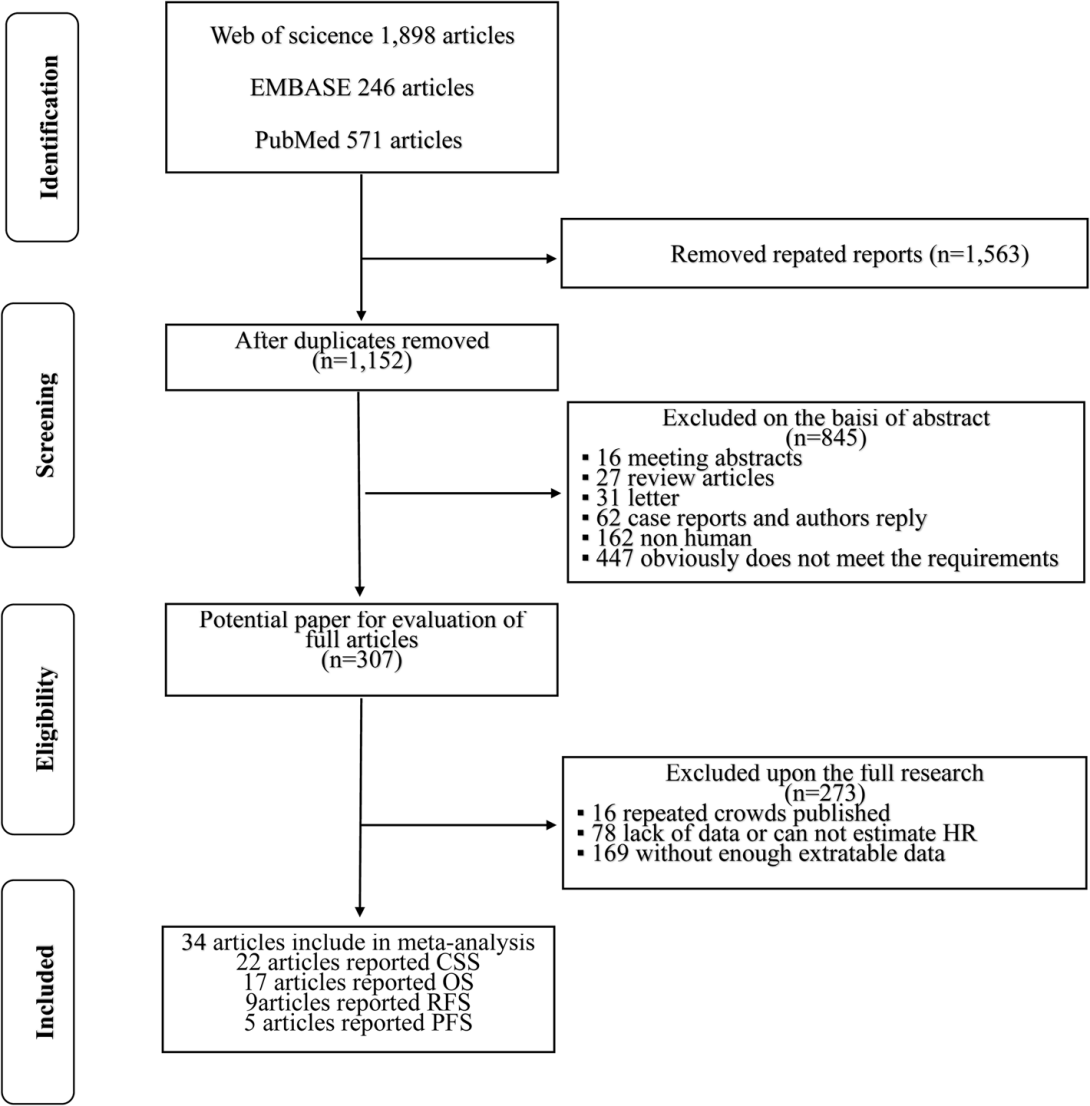
Diagram of the literature search used in this meta-analysis

A diagram of the selection process is shown in Fig. 1. According to the search strategy, 2715 articles were retrieved from the electronic databases. By excluding 1563 duplicate reports, 1152 articles were considered potentially relevant based on screening of the titles and abstracts. The remaining articles were further excluded upon full-text review for several reasons, such as a lack of sufficient data to estimate HRs or duplicate publication in repeated cohorts. Ultimately, 34 studies [3, 12–44] that focused on the association between RCC and TN were included for meta-analysis. The outcomes were CSS in 22 studies, OS in 17 studies, RFS in 9 studies and PFS in 5 studies.

### Characteristics of the included studies

The main characteristics of the 34 eligible studies are listed in Table 1. All of the studies were published between 2005 and 2017, with a mean duration of follow-up varying from 11.7 to 102 months. The present meta-analysis was based on a total sample size of 14,084 patients, ranging from 59 to 3062 patients. The NOS was applied to assess the methodological quality of the included studies, and the results showed that all studies were of high quality (Additional file 1: Table S1). All of the included studies were based on data for retrospective analyses of survival (CSS, OS, RFS, PFS). The characteristics, including tumor features and pathologic outcomes, are summarized in Table 2**.** TN was detected in 31.6% (4452/14,084) of the pathological specimens from the included patients. A total of 13 of the included studies were limited to clear cell renal cell carcinoma (ccRCC), whereas 21 studies involved various tumor types, including ccRCC, papillary renal cell carcinoma, chromophobe renal cell carcinoma and unclassified tumor.

**Table 1:** Main characteristics of the eligible studies

Study	Country	Recruitment period	No. of patients	Age (years)	Gender (m/f)	Follow-up (months)	Study design	Survival analysis	Surgery
Xia et al.2017 [12]	China	2005–2007	293	Median (range) 55 (15–86)	90/203	Median (range) 99.1 (2.63–120.47)	Retrospective	OS,PFS	nephrectomy
Wu et al.2017 [13]	China	2004–2012	301	Median (range) 53 (4–83)	206/95	Median (range) 51.6 (3–121)	Retrospective	OS	nephrectomy
Niu et al.2017 [14]	China	2008–2009	384	Mean ± SD 53.9 ± 14.9	273/111	Median (range) 73 (42–74)	Retrospective	OS,RFS	RN and PN
Kim et al.2017 [15]	Korea	2006–2012	177	Mean ± SD 62 ± 10.9	136/41	Median (range) 19.2 (0.2–63.8)	Retrospective	OS,PFS	nephrectomy
Gu et al.2017 [16]	China	2006–2014	184	Mean ± SD 54.3 ± 13	142/42	Mean ± SD 23.3 ± 14.6	Retrospective	OS, PFS	nephrectomy
Gershman et al.2017 [17]	USA	1980–2010	138	Mean (range) 63 (54–72)	91/47	Median (IQR) 102(67.2–130.8)	Retrospective	CSS, OS	RN and PN
Chen et al.2017 [18]	China	2006–2015	172	Mean ± SD 56.5 ± 12.4	123/40	Mean ± SD 34.4 ± 22.9	Retrospective	CSS,RFS	RN
Chang1 et al.2016 [19]	China	2008–2014	233	Median (IQR) 56(48–62)	170/63	Median (IQR) 68(41–71)	Retrospective	RFS	nephrectomy
Volpe et al.2016 [3]	Italy	2000–2010	308	Median (IQR) 65(57–73)	110/80	Median (IQR) 72(39–108)	Retrospective	CSS	RN
Khor et al.2016 [20]	USA	1985–2003	842	Median(range) 61.5(22.4–89)	527/315	Median (range) 73.2 (0.12–273.6)	Retrospective	OS	RN and PN
NguyenHoang et al.2016 [21]	China	2008–2009	392	Mean ± SD 55.2 ± 12.1	116/276	Median (range) 73 (39–74)	Retrospective	OS, RFS	RN and PN
Errarte et al.2016 [22]	Spain	NA	59	Mean (range) 59 (25–83)	45/14	Mean (range) 65 (1–240)	Retrospective	OS	nephrectomy
Byun et al.2016 [23]	Korea	2000–2014	1284	Mean ± SD 55.9 ± 12.9	913/371	Median (IQR) 39(19–69)	Retrospective	CSS	RN and PN
Huang et al.2015 [24]	China	1991–2011	218	Mean ± SD 58.9 ± 12.2	169/49	Median (IQR) 43(17.8–67.5)	Retrospective	RFS	RN and PN
Cornejo et al.2015 [25]	USA	1984–2010	154	Mean (range) 62.7 (26–86)	125/29	Mean (range) 73.9 (0.13–222)	Retrospective	CSS, OS	RN and PN
Teng et al.2014 [26]	China	2004–2009	378	Mean ± SD 53.4 ± 12.4	272/106	Median (range) 60 (2–97)	Retrospective	CSS, RFS	RN and PN
Park et al.2014 [27]	Korea	2006–2011	83	Mean ± SD 56.3 ± 10.5	60/23	Median (range) 18 (1–62)	Retrospective	OS, PFS	RN and PN
Oliveira et al.2014 [28]	Brazil	1988–2006	94	Mean ± SD 59.7 ± 12.3	67/27	Median 11.7	Retrospective	CSS	RN and PN
Can et al.2014 [29]	Turkey	1995–2012	127	Mean (range) 56 (26–80)	70/57	Mean (range) 46 (3–169)	Retrospective	CSS	RN and PN
Pichler et al.2013 [30]	Austria	2000–2010	994	Mean ± SD 63.2 ± 11.9	599/395	Mean (range) 48.1 (0–132)	Retrospective	CSS, OS	RN and PN
Kruck et al.2013 [31]	Germany	1993–2006	278	Mean ± SD 62.2 ± 12.5	194/84	Median (IQR) 65(20–100)	Retrospective	CSS, OS	RN and PN
Fukatsu et al.2013 [32]	Japan	1986–2008	561	Median(range) 60(21–89)	442/119	Median (range) 55.7 (1–246)	Retrospective	CSS	nephrectomy
Sukov et al.2013 [33]	USA	1970–2002	395	Median(range) 65(25–89)	327/68	NA	Retrospective	CSS	RN and PN
Chang2 et al.2011 [34]	China	2001–2006	328	Mean (range) 59.2 (23–89)	216/112	Mean (range) 46.5 (1.0–97.2)	Retrospective	OS	RN and PN
Leibovich et al.2010 [35]	USA	1970–2003	3062	NA	2,0160/1002	Median (range) 97.2 (0–432)	Retrospective	CSS	RN and PN
Katz et al.2010 [36]	USA	1989–2004	586	Median 61	530/311	Median (range) 61 (1–209)	Retrospective	CSS, OS	RN and PN
Roos et al.2009 [37]	Germany	1990–2006	118	Mean (range) 64.5 (37.8–84.9)	76/42	Median (range) 3.2 (0.3–16.1)	Retrospective	CSS, PFS	nephrectomy
Coons et al.2009 [38]	USA	1988–2006	128	Median(range) 64(35–87)	95/33	Median (range) 25.2 (0–124)	Retrospective	CSS, OS, RFS	nephrectomy
Pflanz et al.2008 [39]	Germany	1992–2006	607	Mean (range) 61.6 (18–84)	387/220	Median 54	Retrospective	CSS, OS	RN and PN
Lee et al.2006 [40]	Korea	1993–2003	485	Median(range) 55(26–81)	360/125	Median(range) 50.9(1–148.6)	Retrospective	CSS	RN and PN
Lam et al.2005 [41]	USA	1989–2000	311	Median(range) 62(27–89)	208/103	Median (range) 45 (0.3–117)	Retrospective	CSS	nephrectomy
Tornberg et al.2016 [42]	Finland	2006–2014	142	Median(range) 65(41–89)	95/47	Median (range) 31 (0–111)	Prospective	CSS	RN and cytoreductive
Schiavina et al.2015 [43]	Italy	2000–2013	185	Mean ± SD 63.3 ± 11.8	149/36	Median (IQR) 32(18–62)	Prospective	CSS	RN and PN
Ramsey et al.2008 [44]	UK	2001–2005	83	NA	50/33	Median 38	Prospective	CSS, RFS	nephrectomy

total numbers rows:36; *SD* standard deviation, *NA* data not applicable, *CSS* cancer-specific survival, *OS* overall survival, *RFS* recurrence-free survival, *PFS* progression-free survival, *RD* radical nephrectomy, *PN* partial nephrectomy

**Table 2:** Tumor characteristics of the eligible studies

Study	Staging system	Grading system	TN+/TN-	Stage 1–2/ 3–4	Grade 1–2/ 3–4	ccRCC/no-ccRCC	Tumor size (cm)
Xia et al.2017 [12]	2010 AJCC	Furman	41/252	212/81	248/45	293/0	NA
Wu et al.2017 [13]	2010 AJCC	Furman	77/224	265/36	225/76	301/0	NA
Niu et al.2017 [14]	2010 AJCC	Furman	75/309	295/89	255/129	384/0	Mean ± SD 4.1 ± 2.1
Kim et al.2017 [15]	2009 AJCC	Furman	46/131	60/82	44/105	159/3	Median (range) 8 (1–117)
Gu et al.2017 [16]	2010 AJCC	Furman	90/94	NA	70/94	161/23	NA
Gershman et al.2017 [17]	2010 AJCC	WHO/ ISUP	111/27	31/106	6/132	105/33	Median (range) 10(8–13)
Chen et al.2017 [18]	2010 AJCC	Furman	53/110	0/163	83/55	135/8	Mean ± SD 6.8 ± 3.5
Chang1 et al.2016 [19]	2010 AJCC	Furman	182/51	169/64	135/96	233/0	NA
Volpe et al.2016 [3]	2002 AJCC	Furman	60/130	190/0	155/35	156/34	Median (IQR) 4.9(3.5–7)
Khor et al.2016 [20]	2010 AJCC	Furman	665/177	630/212	265/577	842/0	Median (range) 4.2(0.6–20)
NguyenHoang et al.2016 [21]	2010 AJCC	Furman	78/294	292/100	259/133	392/0	Mean ± SD 4.3 ± 2.6
Errarte et al.2016 [22]	2010 AJCC	Furman	30/29	32/27	24/35	59/0	Median (range) 7.9(2–19)
Byun et al.2016 [23]	2002 AJCC	Furman	208/1076	1105/179	664/620	1114/170	Mean ± SD 4.08 ± 2.68
Huang et al.2015 [24]	2010 AJCC	Furman	34/184	160/58	155/63	0/218	Median (IQR) 3.5(2.5–6)
Cornejo et al.2015 [25]	NA	Fuhrman/ ISUP	40/114	121/33	103/51	0/154	Mean (range) 5.1(0.4–17)
Teng et al.2014 [26]	2009 AJCC	Furman	38/340	346/32	200/178	378/0	Mean ± SD 4.6 ± 2.6
Park et al.2014 [27]	NA	Furman	37/46	NA	13/70	83/0	NA
Oliveira et al.2014 [28]	2010 AJCC	Furman	18/76	77/17	65/29	94/0	Mean ± SD 4.7 ± 2.6
Can et al.2014 [29]	2010 AJCC	Furman	42/85	84/43	72/55	127/0	NA
Pichler et al.2013 [30]	2010 AJCC	Furman	277/717	723/271	839	804/190	NA
Kruck et al.2013 [31]	2010 AJCC	Furman	114/164	169/109	234/44	278/0	Mean ± SD 5.26 ± 2.91
Fukatsu et al.2013 [32]	2010 AJCC	Furman	57/104	508/53	341/220	561/0	NA
Sukov et al.2013 [33]	2010 AJCC	Furman	186/209	346/49	247/148	0/395	NA
Chang2 et al.2011 [34]	2002 AJCC	Furman	139/189	240/88	216/112	232/96	NA
Leibovich et al.2010 [35]	2002 AJCC	Furman	792/2090	1992/1070	1649/1413	1781/1281	NA
Katz et al.2010 [36]	2002 AJCC	Furman	253/586	575/194	589/252	641/198	NA
Roos et al.2009 [37]	2002 AJCC	Furman	10/108	0/118	63/55	109/16	Median (range) 8(2.5–20)
Coons et al.2009 [38]	2002 AJCC	Furman	57/71	0/128	40/103	105/23	Median (range) 9.9 (3.5–21)
Pflanz et al.2008 [39]	2002WHO	Thoenes	155/452	515/92	532/75	479/128	NA
Lee et al.2006 [40]	1997 AJCC	Furman	131/354	382/103	364/221	419/66	NA
Lam et al.2005 [41]	1997 AJCC	Fuhrman	168/143	157/153	186/119	270/41	NA
Tornberg et al.2016 [42]	2009 AJCC	Furman	84/58	0/132	38/104	129/13	Mean ± SD 10.3 ± 3.6
Schiavina et al.2015 [43]	2009 AJCC	Furman	49/136	0/185	46/139	150/35	Mean ± SD 8.05 ± 2.8
Ramsey et al.2008 [44]	1997 AJCC	Furman	55/28	48/35	37/40	33/50	NA

total numbers rows:36; *TN+/TN* tumor necrosis positive/ tumor necrosis negative, *SD* standard deviation, *NA* data not applicable, *ccRCC/no-ccRCC* clear cell renal cell carcinoma/non- clear cell renal cell carcinoma

### Prognostic value of TN for survival outcome

**Fig. 2:**
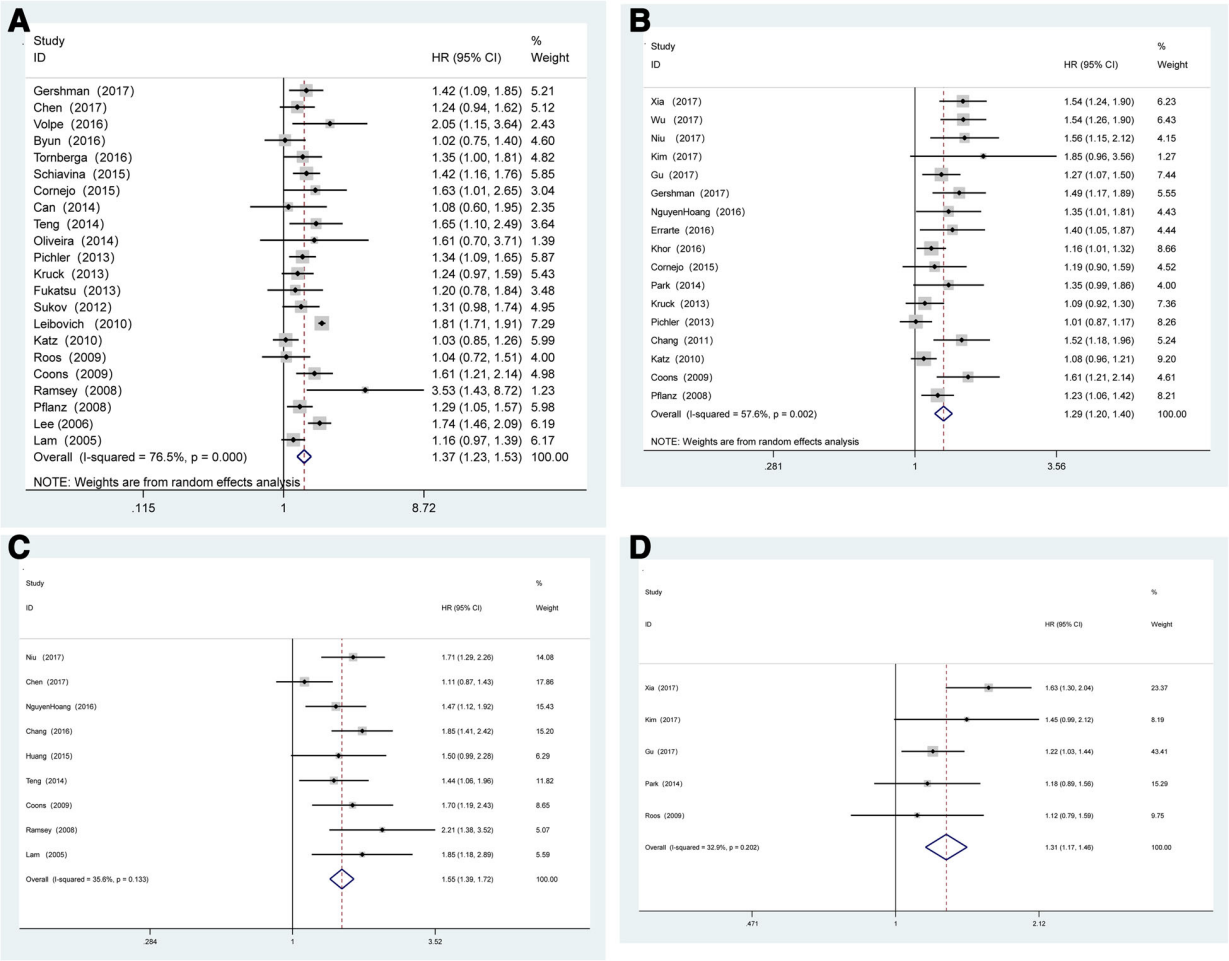
**a** Forest plots of studies evaluating the association between TN and CSS outcomes in RCC patients. **b** Forest plots of studies evaluating the association between TN and OS outcomes in RCC patients. **c** Forest plots of studies evaluating the association between TN and RFS outcomes in RCC patients. **d** Forest plots of studies evaluating the association between TN and PFS outcomes in RCC patients

The present meta-analysis demonstrated that TN in RCC is associated with poor CSS (RE HR = 1.37, 95% CI: 1.23–1.53, *p* < 0.001, ***I***^2^ = 76.5%, P_heterogeneity_ < 0.001; Fig. 2a), OS (RE HR = 1.29, 95% CI: 1.20–1.40, *p* < 0.001, ***I***^2^ = 57.6%, P_heterogeneity_ = 0.02; Fig. 2b), RFS (FE HR = 1.55, 95% CI: 1.39–1.72, *p* < 0.001, **I**^2^ = 35.6%,P_heterogeneity_ = 0.133; Fig. 2c) and PFS (FE HR = 1.31, 95% CI: 1.17–1.46, *p* < 0.001, ***I***^2^ = 32.9%, P_heterogeneity_ = 0.202; Fig. 2d). To explore the source of heterogeneity for CSS, OS and RFS, subgroup analysis was conducted according to geographical region (Asia vs. other regions), pathological type (ccRCC vs. other types), staging system (2010 AJCC vs. other system), No. of patients (≥ 300 vs. < 300) and median follow-up (≥ 40 months vs. < 40 months). The results of this subgroup analysis again suggested that TN is a prognostic factor, despite heterogeneity among some groups (Table 3). Notably, heterogeneity for CSS, OS and RFS was significantly decreased in some models, such as geographical region in Asia, ccRCC pathological type, 2010 AJCC staging system and ≥ 300 cases.

**Table 3:** Summary and subgroup analysis for the eligible studies

Analysis specification	No. of studies	Study heterogeneity		Effects model	Pooled HR(95% CI)	*p*-Value
		I^2^ (%)	P_heterogeneity_			
CSS						
Overall	22	76.5	< 0.001	Random	1.37(1.23,1.53)	< 0.001
Geographical region						
Asian	7	51.7	0.053	Random	1.34(1.12,1.59)	0.001
Other regions	15	80.8	< 0.001	Random	1.40(1.22,1.60)	< 0.001
Pathological types						
ccRCC	6	0	0.775	Fixed	1.34(1.15,1.55)	< 0.001
Other types	16	81.7	< 0.001	Random	1.38(1.22,1.58)	< 0.001
Staging system						
2010 AJCC	8	0	0.981	Fixed	1.30(1.17,1.44)	< 0.001
Other system	14	82.3	< 0.001	Random	1.42(1.23,1.64)	< 0.001
No. of patients						
≥ 300	13	82	< 0.001	Random	1.39(1.21,1.61)	< 0.001
< 300	9	15,4	0.301	Fixed	1.33(1.16,1.51)	< 0.001
Median follow-up						
≥ 40 months	12	83.3	< 0.001	Random	1.36(1.16,1.60)	< 0.001
< 40 months	9	30.2	0.177	Fixed	1.33(1.16,1.51)	< 0.001
OS						
Overall	17	57.6	0.002	Random	1.29(1.20,1.40)	< 0.001
Geographical region						
Asian	9	30.2	0.177	Fixed	1.38(1.25,1.51)	< 0.001
Other regions	8	58.6	0.017	Random	1.20(1.09,1.34)	< 0.001
Pathological types						
ccRCC	8	48.8	0.057	Random	1.33(1.19,1.49)	< 0.001
Other types	9	62.7	0.006	Random	1.26(1.13,1.41)	< 0.001
Staging system						
2010 AJCC	10	63.6	0.003	Random	1.30(1.17,1.44)	< 0.001
Other system	7	53.1	0.046	Random	1.30(1.14,1.47)	< 0.001
No. of patients						
≥ 300	8	67.5	0.003	Random	1.25(1.12,1.39)	< 0.001
< 300	9	29.2	0.185	Fixed	1.35(1.22,1.49)	< 0.001
Median follow-up						
≥ 40 months	13	62.6	0.001	Random	1.27(1.16,1.39)	< 0.001
< 40 months	4	0	0.412	Fixed	1.37(1.20,1.56)	< 0.001
RFS						
Overall	9	35.6	0.133	Fixed	1.55(1.39,1.72)	< 0.001
Geographical region						
Asian	6	42.7	0.12	Fixed	1.48(1.31,1.66)	< 0.001
Other regions	3	0	0.684	Fixed	1.87(1.41,2.37)	< 0.001
Pathological types						
ccRCC	4	0	0.541	Fixed	1.61(1.40,1.86)	< 0.001
Other types	5	57.5	0.051	Random	1.46(1.25,1.71)	< 0.001
Staging system						
2010 AJCC	5	54	0.069	Random	1.48(1.31,1.69)	< 0.001
Other system	4	0	0.483	Fixed	1.69(1.40,2.04)	< 0.001
No. of patients						
≥ 300	4	0	0.702	Fixed	1.57(1.35,1.83)	< 0.001
< 300	5	63.4	0.027	Random	1.52(1.32,1.76)	< 0.001
Median follow-up						
≥ 40 months	6	0	0.758	Fixed	1.62(1.43,1.84)	< 0.001
< 40 months	3	75.3	0.018	Random	1.39(1.16,1.68)	0.001
PFS						
Overall	5	32.9	0.202	Fixed	1.31(1.17,1.46)	< 0.001
Pathological types						
ccRCC	2	67.8	0.078	Random	1.44(1.20,1.71)	< 0.001
Other types	3	0	0.6	Fixed	1.23(1.07,1.41)	0.004
Staging system						
2010 AJCC	2	76.3	0.04	Random	1.35(1.18,1.54)	< 0.001
Other system	3	0	0.582	Fixed	1.22(1.01,1.48)	0.036

### Sensitivity analyses and publication bias

**Fig. 3:**
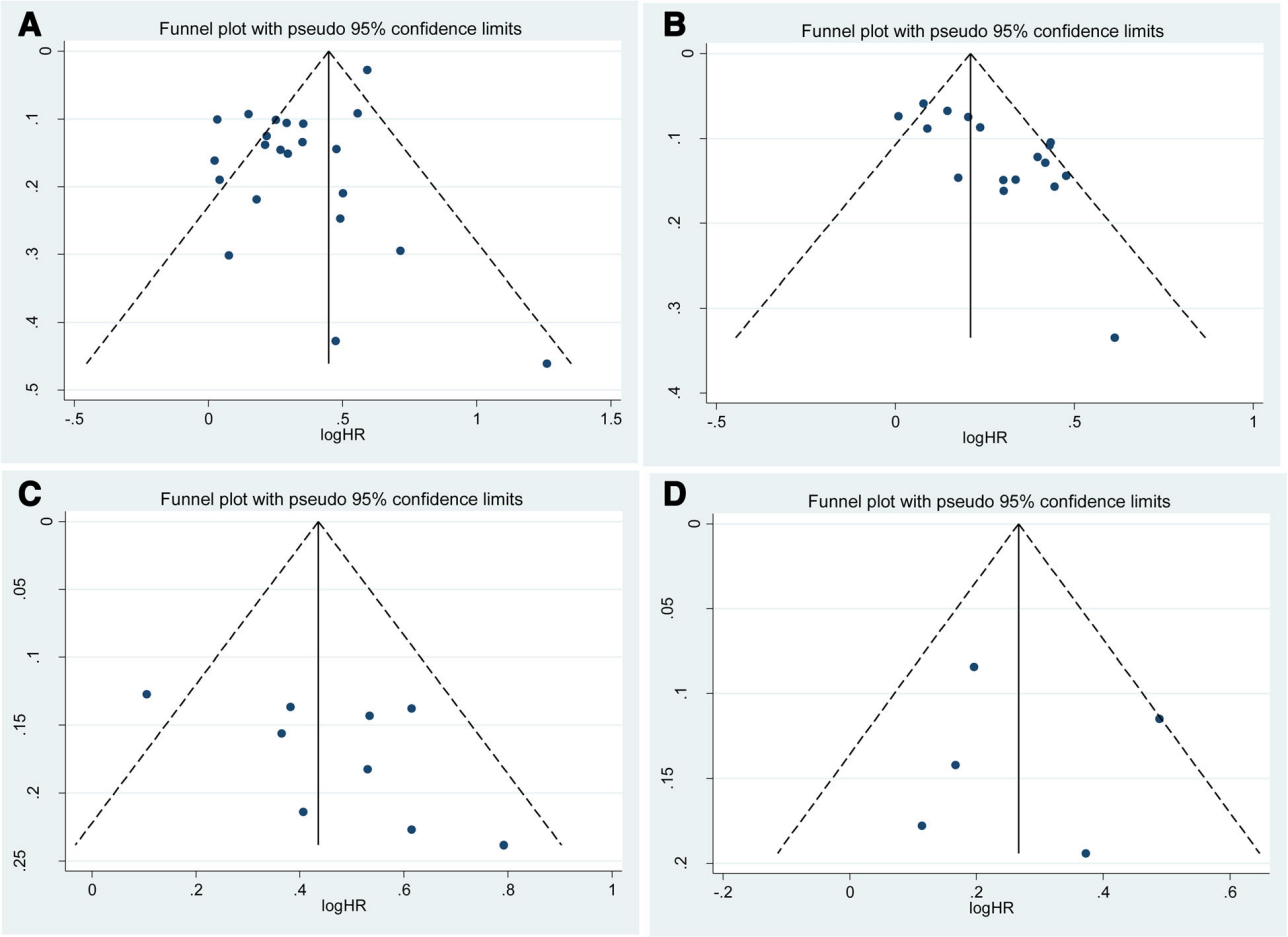
**a** Funnel plots and Egger’s tests for the publication bias of CSS in hazard ratios (HRs). **b** Funnel plots and Egger’s tests for the publication bias of OS in hazard ratios (HRs). **c** Funnel plots and Egger’s tests for the publication bias of RFS in hazard ratios (HRs). **d** Funnel plots and Egger’s tests for the publication bias of PFS in hazard ratios (HRs)

In sensitivity analysis excluding one study at a time, the pooled HR for CSS ranged from 1.29 (95% CI: 1.19–1.39) to 1.37 (95% CI: 1.22–1.54) (Additional file 2: Figure S1). Similarly, the pooled HR for OS ranged from 1.27 (95% CI: 1.17–1.37) to 1.31 (95% CI: 1.21–1.42) (Additional file 3: Figure S2), that for RFS from 1.52 (95% CI:1.32–1.76) to 1.66 (95% CI: 1.47–1.86) (Additional file 4: Figure S3), and that for PFS from 1.21 (95% CI:1.07–1.38) to 1.35 (95% CI: 1.12–1.63) (Additional file 5: Figure S4). These results indicate that the findings were reliable and robust. Although no statistical evidence of publication bias was observed for RFS (p-Egger = 0.135, Fig. 3c) and PFS (p-Egger = 0.932, Fig. 3d), publication bias was observed for CSS (p-Egger = 0.006, Fig. 3a) and OS (p-Egger = 0.001, Fig. 3b).

## Discussion

RCC is the most common solid lesion of the kidney, and more than 40% of patients die from this type of cancer [2]. Despite significant improvements in systemic therapy for RCC, the prognosis of patients with RCC and treatment response rates have not substantially increased [17, 42, 44]. Although several pathologic parameters, including lymphatic vessel invasion [45], tumor fat invasion [26] and primary tumor size [43], provide independent prognostic information, the likely outcome for an individual patient remains uncertain. The TNM stage and Fuhrman grade system are the most widely used approaches for RCC; however, there have been many recent suggestions for modifications based on survival trends in large case series [46]. Additionally, RCC is a highly heterogeneous disease with different clinical presentations and characteristics that remain somewhat unpredictable [47]. Therefore, it is essential to optimize the treatment and prognosis of RCC and to provide better counseling for each RCC patient.

The presence of TN in pathologic specimens may reflect the tumor biology and may also provide additional useful prognostic information. As TN results from rapid tumor proliferation and consequent outgrowth of the blood supply [41], histologic TN has been proposed to be a sign of tumor aggressiveness that generally leads to poor clinical outcomes [48]. Previous studies have investigated the association of TN with various solid tumors, including breast cancer [49], colorectal cancer [50] and lung cancer [51]. Indeed, there is renewed interest in using TN, which can be assessed in every routine pathological examination without additional costs, to more accurately predict the clinical outcome of RCC. For example, Khor et al. [20] and Ito et al. [48] reported that TN is strongly associated with poor survival and should serve as an independent prognostic factor for patients with RCC. Nonetheless, some studies have shown that the presence of any TN is a negative predictor of survival in RCC [52, 53].

To our knowledge, the present study is the first meta-analysis on the association between TN and clinical outcomes of different types of RCC. In this analysis, 14,084 RCC patients were included from 34 cohort studies, and TN was detected in 31.6% of 4452 RCC patients. Robust evidence obtained from sensitivity analysis demonstrated that the presence of TN was associated with poor outcomes in terms of CSS (HR = 1.37, *p* < 0.001), OS (HR = 1.29, *p* < 0.0 01), RFS (HR = 1.55, *p* < 0.001) and PFS (HR = 1.31, *p* < 0.001) in patients with RCC. These findings were consistently independent of geographical region, pathological type, staging system, No. of patients and median follow-up. Although there was no evidence of heterogeneity in terms of CSS or PFS, significant heterogeneity was detected in analyses of OS and RFS models. To further explore the source of heterogeneity in OS and RFS, subgroup analysis was conducted, and the data showed that significant variations were reduced in OS and RFS within some items.

Notably, the present study has several limitations. First, all the included studies were retrospective cohort studies, and data extracted from those studies may have led to inherent potential bias. Second, the criteria for determining the presence of TN in a pathologic specimen were inconsistent in the included studies, which may contribute to heterogeneity. Thus, rigorous morphological criteria should be used to standardize the diagnosis of TN. Third, we only included published studies written in English, and the lack of “gray literature” may cause selection bias. Fourth, substantial heterogeneity was observed in meta-analysis of CSS and OS, and although we selected the RE model according to heterogeneity, this diversity remained. Using subgroup analysis, we propose that the heterogeneity likely reflected differences in factors, such as patient and tumor characteristics. Fifth, a statistical publication bias was observed for CSS and OS according to Egger’s test. In general, studies with negative results tend not to be submitted or published; therefore, a certain degree of publication bias was observed in the present study. Finally, it should be noted that factors, including age, sex, histology type and surgical method, that may affect survival outcomes were adequately controlled.

Nevertheless, the present study has several key strengths. First, the meta-analysis included 34 studies with large sample sizes, with the ability to detect more stable associations between TN and clinical outcomes of RCC patients. Second, with strict inclusion and exclusion criteria, we extracted available data from relevant studies. Furthermore, through subgroup and sensitivity analyses, the results were reliable and robust. Therefore, TN determination, with excellent accessibility and low costs, warrants wider application in patients with RCC for risk stratification and decision-making of individualized treatment.

## Conclusions

In conclusion, the results of the present meta-analysis demonstrate that TN in histopathology is associated with poor CSS, OS, RFS and PFS in patients with RCC. Due to the limitations of the present study, large-scale, multicenter prospective studies with long-term follow-up are needed to verify these results.

## Additional files

Additional file 1: **Table S1.** Quality assessment of cohort studies included in this meta- analysis. (DOCX 15 kb)

Additional file 2: **Figure S1.** Sensitivity analysis of the association between TN and CSS outcomes in RCC patients. (TIF 302 kb)

Additional file 3: **Figure S2.** Sensitivity analysis of the association between TN and OS outcomes in RCC patients. (TIF 255 kb)

Additional file 4: **Figure S3.** Sensitivity analysis of the association between TN and RFS outcomes in RCC patients. (TIF 157 kb)

Additional file 5: **Figure S4.** Sensitivity analysis of the association between TN and PFS outcomes in RCC patients. (TIF 115 kb)

## Abbreviations

AJCC: American Joint Committee on Cancer
ccRCC: Clear cell renal cell carcinoma
CIs: Corresponding 95% confidence intervals
CSS: Cancer-specific survival
FE: Fixed-effects
HRs: Hazard ratios
ISUP: International Society of Urologic Pathologists
NOS: Newcastle Ottawa scale
OS: Overall survival
PFS: Progression-free-survival
PRISMA: Preferred Reporting Items for Systematic Reviews and Meta-Analyses
RCC: Renal cell carcinoma
RE: Random-effects
RFS: Recurrence-free survival
SSIGN: Mayo Clinic Stage, Size, Grade and Necrosis
TN: Tumor necrosis
