# Supplementary material for: Tumor necrosis as a prognostic variable for the clinical outcome in patients with renal cell carcinoma: a systematic review and meta-analysis
Source: BMC Cancer. 2018 Sep 3;18:870. doi: 10.1186/s12885-018-4773-z (PMC6122538; doi:10.1186/s12885-018-4773-z)
Supplement: Supplementary file 1 — Table S1. Quality assessment of cohort studies included in this meta- analysis. (DOCX 15 kb) [file 12885_2018_4773_MOESM1_ESM.docx]

**Table S1. Quality assessment of cohort studies included in this meta- analysis**

| **Study** | **Representativeness of the exposed cohort** | **Selection of the unexposed cohort** | **Ascertainment of exposure** | **Outcome of interest not present at start of study** | **Control for important factor or additional factor** | **Outcome assessment** | **Follow-up long enough for outcomes to occur** | **Adequacy of follow-up of cohort** | **Total quality scores** |
| --- | --- | --- | --- | --- | --- | --- | --- | --- | --- |
| Xia et al.2017 | ★ | ★ | ★ | ★ | ★ | ★ | ★ | ★ | 8 |
| Wu et al.2017 | ★ | ★ | ★ | ★ | ★ | ★ | ★ | ★ | 8 |
| Niu et al.2017 | ★ | ★ | ★ | ★ | ★★ | ★ | ★ | ★ | 9 |
| Kim et al.2017 | ★ | ★ | — | ★ | ★ | ★ | ★ | ★ | 7 |
| Gu et al.2017 | ★ | ★ | ★ | ★ | ★★ | ★ | ★ | ★ | 9 |
| Gershman et al.2017 | ★ | ★ | ★ | ★ | ★★ | ★ | ★ | ★ | 9 |
| Chen et al.2017 | ★ | ★ | ★ | ★ | ★★ | ★ | ★ | ★ | 9 |
| Chang1 et al.2016 | ★ | ★ | ★ | ★ | ★★ | ★ | ★ | ★ | 9 |
| Volpe et al.2016 | ★ | ★ | ★ | ★ | ★ | ★ | ★ | ★ | 8 |
| Khor et al.2016 | ★ | ★ | — | ★ | ★★ | ★ | ★ | ★ | 8 |
| NguyenHoang et al.2016 | ★ | ★ | ★ | ★ | ★★ | ★ | ★ | ★ | 9 |
| Errarte et al.2016 | ★ | ★ | — | ★ | ★ | ★ | ★ | ★ | 7 |
| Byun et al.2016 | ★ | ★ | ★ | ★ | ★ | ★ | ★ | ★ | 8 |
| Huang et al.2015 | ★ | ★ | ★ | ★ | ★ | ★ | ★ | ★ | 8 |
| Cornejo et al.2015 | ★ | ★ | ★ | ★ | ★★ | ★ | ★ | ★ | 9 |
| Teng et al.2014 | ★ | ★ | ★ | ★ | ★ | ★ | ★ | ★ | 8 |
| Park et al.2014 | ★ | ★ | ★ | ★ | ★ | ★ | ★ | ★ | 8 |
| Oliveira et al.2014 | ★ | ★ | — | ★ | ★★ | ★ | ★ | ★ | 8 |
| Can et al.2014 | ★ | ★ | — | ★ | ★ | ★ | ★ | ★ | 7 |
| Pichler et al.2013 | ★ | ★ | ★ | ★ | ★ | ★ | ★ | ★ | 8 |
| Kruck et al.2013 | ★ | ★ | ★ | ★ | ★ | ★ | ★ | ★ | 8 |
| Fukatsu et al.2013 | ★ | ★ | ★ | ★ | ★★ | ★ | ★ | ★ | 9 |
| Sukov et al.2013 | ★ | ★ | — | ★ | ★★ | ★ | ★ | ★ | 8 |
| Chang2 et al.2011 | ★ | ★ | ★ | ★ | ★★ | ★ | ★ | ★ | 9 |
| Leibovich et al.2010 | ★ | ★ | ★ | ★ | ★ | ★ | — | ★ | 7 |
| Katz et al.2010 | ★ | ★ | ★ | ★ | ★★ | ★ | ★ | ★ | 9 |
| Roos et al.2009 | ★ | ★ | ★ | ★ | ★ | ★ | — | ★ | 7 |
| Coons et al.2009 | ★ | ★ | ★ | ★ | ★★ | ★ | ★ | ★ | 9 |
| Pflanz et al.2008 | ★ | ★ | ★ | ★ | ★ | ★ | ★ | ★ | 8 |
| Lee et al.2006 | ★ | ★ | ★ | ★ | ★ | ★ | ★ | ★ | 8 |
| Lam et al.2005 | ★ | ★ | ★ | ★ | ★ | ★ | ★ | ★ | 8 |
| Tornberg et al.2016 | ★ | ★ | ★ | ★ | ★★ | ★ | ★ | ★ | 9 |
| Schiavina et al.2015 | ★ | ★ | ★ | ★ | ★ | ★ | ★ | ★ | 8 |
| Ramsey et al.2008 | ★ | ★ | ★ | ★ | ★★ | ★ | ★ | ★ | 9 |
