# Supplementary figures and images for: Tumor necrosis as a prognostic variable for the clinical outcome in patients with renal cell carcinoma: a systematic review and meta-analysis
Source: BMC Cancer. 2018 Sep 3;18:870. doi: 10.1186/s12885-018-4773-z (PMC6122538; doi:10.1186/s12885-018-4773-z)

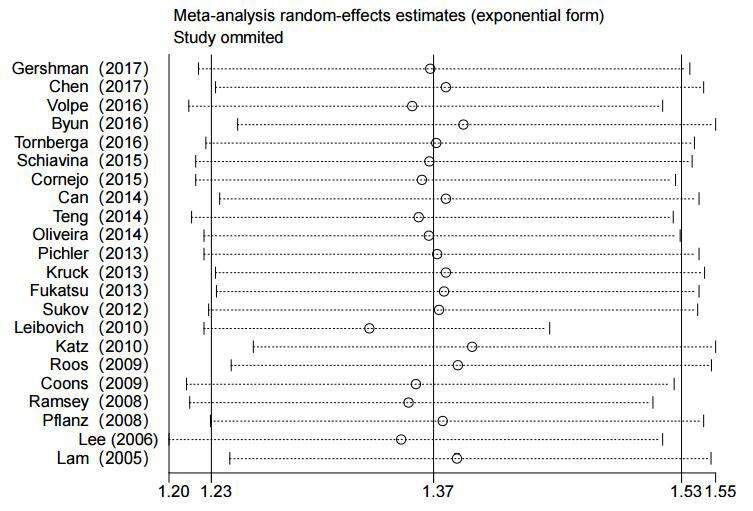

Supplement: Supplementary file 2 — Figure S1. Sensitivity analysis of the association between TN and CSS outcomes in RCC patients. (TIF 302 kb) [file 12885_2018_4773_MOESM2_ESM.tif]

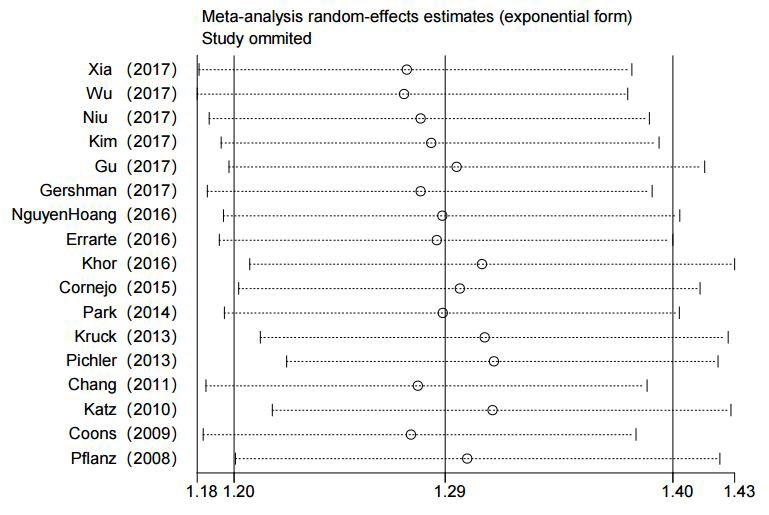

Supplement: Supplementary file 3 — Figure S2. Sensitivity analysis of the association between TN and OS outcomes in RCC patients. (TIF 255 kb) [file 12885_2018_4773_MOESM3_ESM.tif]

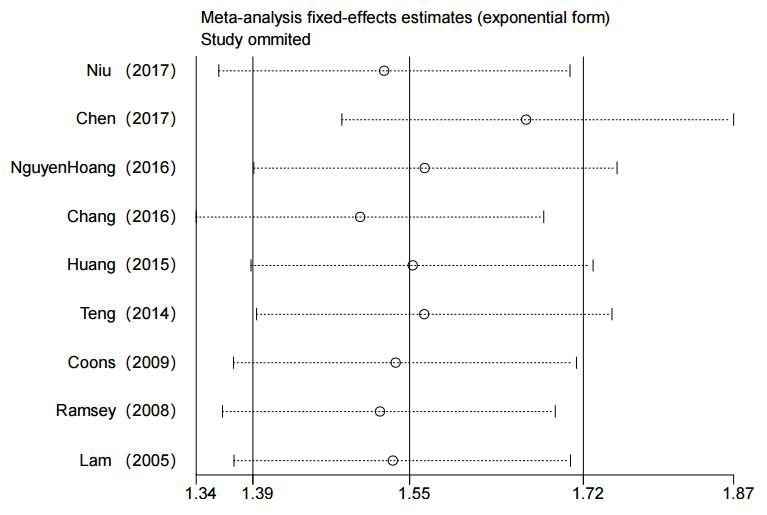

Supplement: Supplementary file 4 — Figure S3. Sensitivity analysis of the association between TN and RFS outcomes in RCC patients. (TIF 157 kb) [file 12885_2018_4773_MOESM4_ESM.tif]

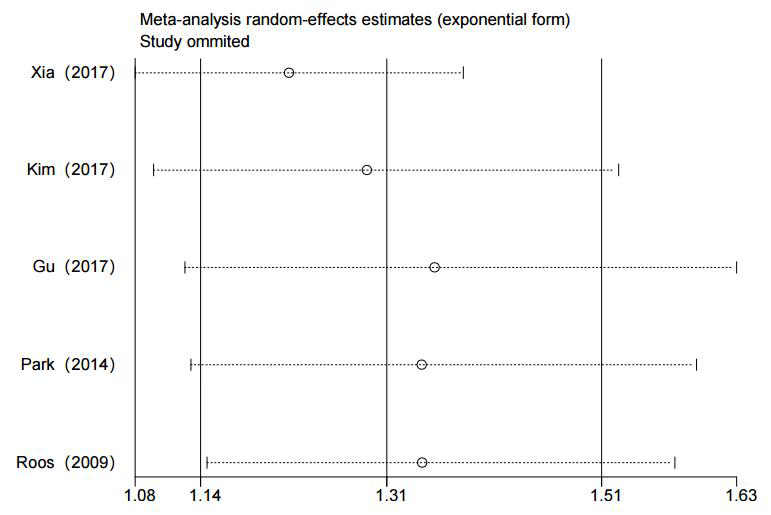

Supplement: Supplementary file 5 — Figure S4. Sensitivity analysis of the association between TN and PFS outcomes in RCC patients. (TIF 115 kb) [file 12885_2018_4773_MOESM5_ESM.tif]
